# Self Protection from Anti-Viral Responses – Ro52 Promotes Degradation of the Transcription Factor IRF7 Downstream of the Viral Toll-Like Receptors

**DOI:** 10.1371/journal.pone.0011776

**Published:** 2010-07-26

**Authors:** Rowan Higgs, Elisa Lazzari, Claire Wynne, Joan Ní Gabhann, Alexander Espinosa, Marie Wahren-Herlenius, Caroline A. Jefferies

**Affiliations:** 1 Molecular and Cellular Therapeutics and RSCI Research Institute, Royal College of Surgeons in Ireland, Dublin, Ireland; 2 Department of Medicine, Karolinska Institutet, Stockholm, Sweden; New York University, United States of America

## Abstract

Ro52 is a member of the TRIM family of single-protein E3 ligases and is also a target for autoantibody production in systemic lupus erythematosus and Sjögren's syndrome. We previously demonstrated a novel function of Ro52 in the ubiquitination and proteasomal degradation of IRF3 following TLR3/4 stimulation. We now present evidence that Ro52 has a similar role in regulating the stability and activity of IRF7. Endogenous immunoprecipitation of Ro52-bound proteins revealed that IRF7 associates with Ro52, an effect which increases following TLR7 and TLR9 stimulation, suggesting that Ro52 interacts with IRF7 post-pathogen recognition. Furthermore, we show that Ro52 ubiquitinates IRF7 in a dose-dependent manner, resulting in a decrease in total IRF7 expression and a subsequent decrease in IFN-α production. IRF7 stability was increased in bone marrow-derived macrophages from Ro52-deficient mice stimulated with imiquimod or CpG-B, consistent with a role for Ro52 in the negative regulation of IRF7 signalling. Taken together, these results suggest that Ro52-mediated ubiquitination promotes the degradation of IRF7 following TLR7 and TLR9 stimulation. As Ro52 is known to be IFN-inducible, this system constitutes a negative-feedback loop that acts to protect the host from the prolonged activation of the immune response.

## Introduction

A critical step in the innate immune response to viral infection is the production of type I IFN (IFN-α and -β) [1]. In particular, the regulatory networks that regulate that activity of the IFN regulatory factor (IRF) family members IRF3 and IRF7 have been an area of intense study of late [2], [3], [4], [5], [6]. Indeed, a number of endogenous and viral proteins have recently been described which positively or negatively regulate the activity of IRF family members, thus manipulating the type I IFN response [7], [8], [9], [10], [11], [12], [13].

The cellular mechanisms involved in IRF7-mediated type I IFN gene induction have been studied intensely in recent years. The ubiquitin E3 ligase TNF receptor-associated factor 6 (TRAF6) is known to ubiquitinate IRF7 in a K63-dependent manner following TLR stimulation, resulting in IRF7 activation and the initiation of IFN-α transcription [14]. This effect has been shown to depend on the formation of a complex between IRF7, TRAF6 and MyD88 and is critical for type I IFN production downstream of TLR7, TLR8 and TLR9 stimulation [14], [15]. Furthermore, the E3 ligase activity of TRAF6 is also known to be required for latent membrane protein 1 (LMP1)-stimulated IRF7 ubiquitination and may be a prerequisite for IRF7 phosphorylation by IKKα, IKKε, TANK-binding kinase 1, or IL-1 receptor-associated kinase 1 [16], [17], [18]. Following IRF7-mediated induction of type I IFNs, it is known that IRF7 is rapidly degraded in order to protect the host from excessive production of IFNs [19]. However, the endogenous E3 ligase responsible for initiating the degradation of IRF7 as part of the normal immune response has not yet been identified.

Ro52 (also denoted TRIM21 and SSA1) is a member of the tripartite motif (TRIM) family of single-protein E3 ligases and is known to be a target for autoantibody production in systemic lupus erythematosus and Sjögren's syndrome [20], [21], [22], [23], [24], [25]. Ro52 expression is known to IFN-inducible and through several recent studies, is emerging as a critical regulator of IRF stability. Work performed in our lab described a novel function of Ro52 in the negative regulation of IFN-β production following TLR3/4 stimulation. Ro52 achieves this by promoting the ubiquitination and proteasomal degradation of IRF3 [13]. Furthermore, through the generation of Ro52-deficient mice, Ro52 has recently been implicated in a type I IFN negative feedback loop, responsible for limiting the production of type I IFNs and for preventing the onset of an IL-23p19-mediated lupus-like disease [26].

To date the endogenous cellular mechanism to turn off IRF7 signalling has not been described. In this study we provide the first evidence that Ro52 and IRF7 associate endogenously, leading to the ubiquitination and subsequent degradation of IRF7. These results are consistent with the reported effects of Ro52 deficiency in mice, thus adding further evidence that Ro52 functions as part of a negative feedback loop to protect the host from the deleterious effects of prolonged type I IFN production.

## Results

### Ro52 associates endogenously with IRF7

**Figure 1:**
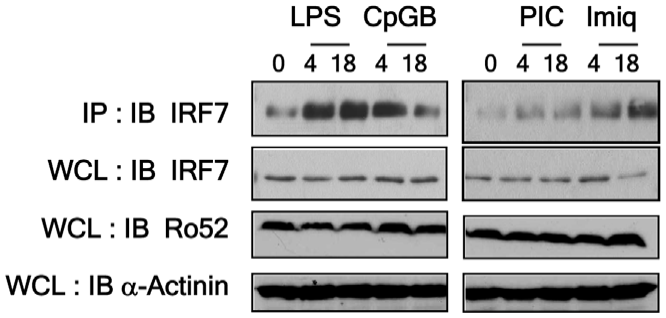
Ro52 interacts with IRF7. HeLa cells were stimulated with the indicated ligands for 4 and 18 hrs. Cell lysates were immunoprecipitated with an anti-Ro52 antibody and immunoblotted with an anti-IRF7 antibody, an anti-Ro52 antibody and an anti-α-actinin antibody.

We have previously shown that recombinant Ro52 interacts with both overexpressed IRF3 and IRF7 in a pulldown experiment [13]. In order to confirm the association of Ro52 with endogenous IRF7 and to examine the status of this relationship following TLR stimulation, HeLa cells were stimulated with either polyI:C, lipopolysaccharide (LPS), imiquimod or CpG-B for 4 and 18 hours. Cell lysates were immunoprecipitated with an anti-Ro52 antibody and IRF7 association was detected by immunoblotting with an anti-IRF7 antibody (Figure 1). A weak endogenous association was observed in unstimulated cells, confirming our previous result [13]. Interestingly, this interaction increased following LPS, imiquimod and CpG-B stimulation and to a lesser extent following 2 hours polyI:C stimulation. The interaction between Ro52 and IRF7 was maintained at 18 hrs LPS stimulation, whereas following imiquimod stimulation, the association between Ro52 and IRF7 was observed to increase at 18 hours. By contrast, a decrease in Ro52-IRF7 interaction was observed at 18 hrs CpG-B stimulation. Importantly, the changes observed in Ro52-IRF7 interaction were not due to fluctuations in either Ro52 or IRF7 levels in the cells as demonstrated in Figure 1. This results suggests that Ro52 targets IRF7 following TLR stimulation and that the longevity of this interaction is TLR-specific.

### Ro52 ubiquitinates IRF7 in a dose-dependent manner

**Figure 2:**
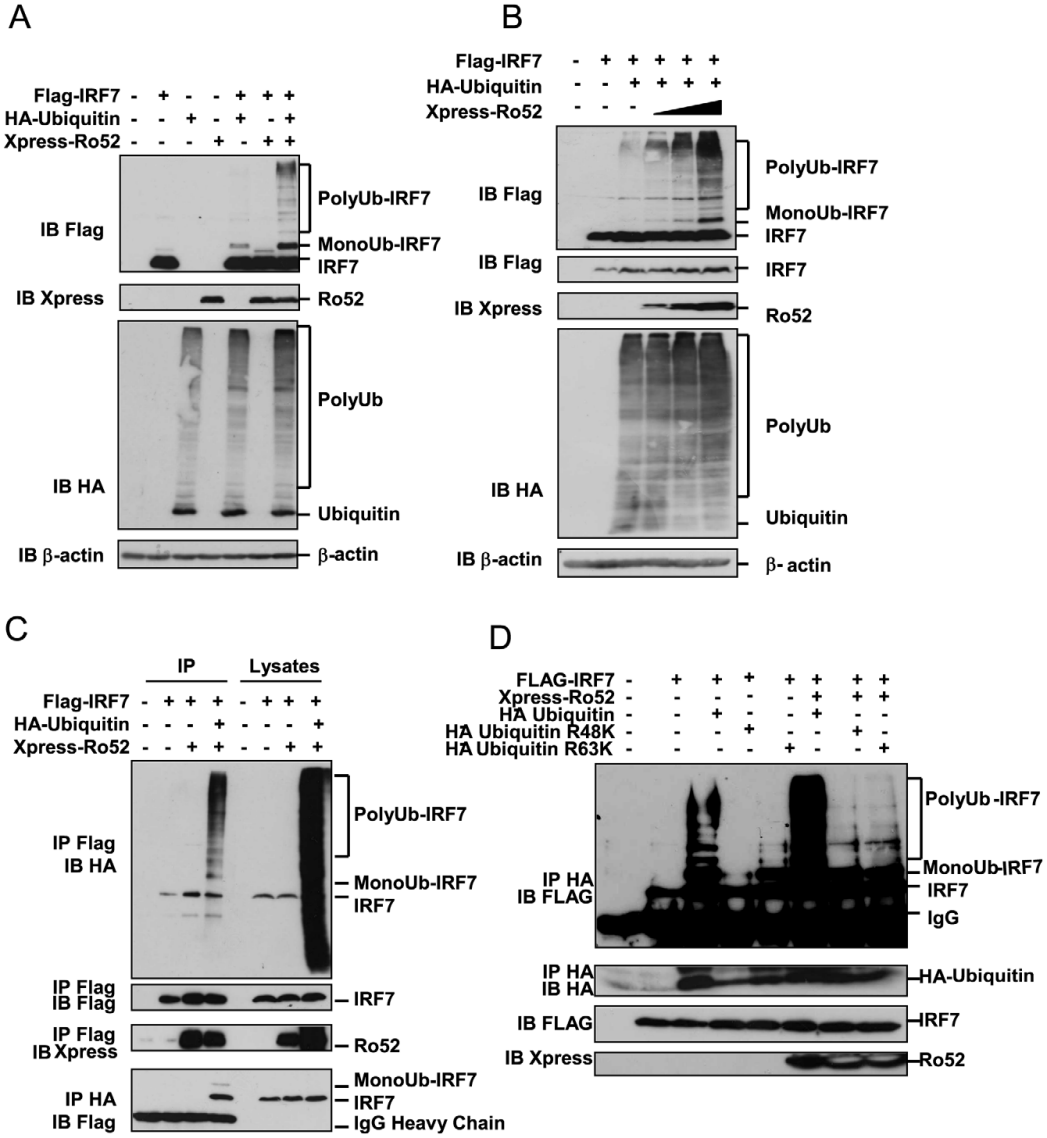
Ro52 ubiquitinates IRF7. (A) HEK293T cells were transfected with 1 µg of the indicated constructs for 18 hrs. Presence of Flag-IRF7, HA-ubiquitin, Xpress-Ro52 and β-actin was detected by immunoblotting. (B) HEK293T cells were transfected with 500 ng of Flag-IRF7, 1 µg of HA-ubiquitin and increasing amounts of Xpress-Ro52 (500 ng, 1 µg and 2 µg) for 18 hrs. Presence of Flag-IRF7, HA-ubiquitin, Xpress-Ro52 and β-actin was detected by immunoblotting. (C) HEK293T cells were transfected with 500 ng of Flag-IRF7, 1 µg of HA-ubiquitin and 500 ng of Xpress-Ro52 for 18 hrs. Flag-IRF7-bound proteins and HA-ubiquitinated proteins were immunoprecipitated from HEK293T cell lysates with anti-Flag and anti-HA antibodies. Presence of HA-ubiquitinated Flag-IRF7 was detected by immunoblotting. (D) HEK293T cells were transfected with 500 ng of Flag-IRF7, 1 µg of HA-ubiquitin plasmids (both WT and mutants) and 500 ng of Xpress-Ro52 for 18 hrs. Flag-IRF7-bound proteins and HA-ubiquitinated proteins were immunoprecipitated from HEK293T cell lysates with anti-HA antibodies. Presence of HA-ubiquitinated Flag-IRF7, HA-ubiquitin, and Xpress-Ro52 was detected by immunoblotting.

As Ro52 is known to ubiquitinate both IRF3 and IRF8, we next examined the potential of Ro52 to ubiquitinate IRF7 in a co-transfection experiment. Human embryonic kidney (HEK) 293T cells were transfected with plasmids expressing Flag-IRF7, Xpress-Ro52 and hemagglutinin (HA)-ubiquitin and cell lysates were immunoblotted with various antibodies. Our results clearly show that IRF7 is polyubiquitinated only when it is co-expressed with Ro52 and HA-ubiquitin (Figure 2A). Increasing amounts of Ro52 resulted in a dose-dependent increase in IRF7 ubiquitination (Figure 2B), indicating that this effect is directly regulated by Ro52. These results demonstrate the ability of Ro52 to add ubiquitin moieties to IRF7, and combined, these results strongly suggest that Ro52 targets IRF7 for ubiquitination following TLR stimulation.

To confirm that the observed higher molecular weight bands were indeed ubiquitinated forms of IRF7, HEK293T cells were transfected with plasmids expressing Flag-IRF7, Xpress-Ro52 and HA-ubiquitin and cell lysates were immunoprecipitated with both anti-Flag and anti-HA antibodies. Following immunoprecipitation of Flag-IRF7-bound proteins, an immunoblot was performed for Flag to confirm the expression of IRF7 and the ability of the Flag antibody to successfully bind to the IgG sepharose beads (Fig. 2C, *second panel, lanes 2–4*). The same membrane was then immunoblotted for HA-ubiquitin and revealed the presence of polyubiquitinated IRF7 only in the lane expressing both ubiquitin and Ro52 (Fig. 2C, *upper panel, lane 4*). This effect was also observed by immunoprecipitating HA-ubiquitin bound proteins followed by a Flag-IRF7 immunoblot (Fig. 2C, *lower panel, lane 4*). Together our results clearly show that IRF7 and Ro52 interact following TLR stimulation and that this interaction serves as a means to polyubiquitinate IRF7.

During the ubiquitinaiton process, the specific lysine on the ubiquitin molecule used by the E3 ligase to attach it to the target protein often determines the fate of the protein. To determine whether Ro52 ubiquitinates IRF7 in a K48- or K63-dependent manner, specific ubiquitin mutants were used, in which all lysines in the ubiquitin sequence were mutated to arginine, with the exception of either K48 (ubiquitin R48K mutant) or K63 (ubiquitin R63K mutant). Co-expression of Flag-IRF7, Xpress-Ro52 and either HA-ubiquitin R48K or HA-ubiquitin R63K resulted in a considerable decrease in IRF7 polyubiquitination in comparison to full length HA-ubiquitin, suggesting that both the K48 and K63 residues are involved in the Ro52-mediated polyubiquitination of IRF7 (Figure 2D). Interestingly, in the absence of Ro52, ubiquitination of IRF7 was not observed when expressed with R48K, suggesting that Ro52 may be important in mediating K48-linked polyubiquitination of IRF7. As expected, the presence of overexpressed Ro52 markedly increased IRF7 polyubiquitination, although IRF7 was still ubiquitinated by endogenous E3 ligases (which include Ro52).

### Ro52 inhibits IRF7-driven IFNα4-reporter gene activation

**Figure 3:**
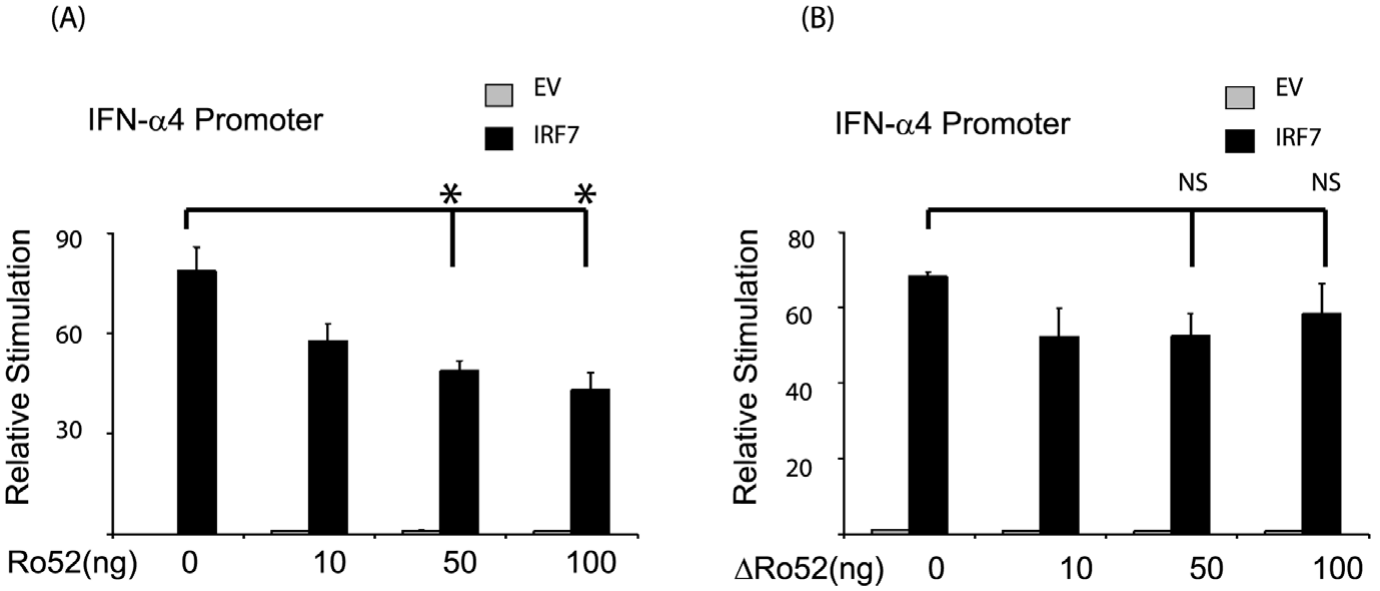
Ro52 inhibits IFN-α4 reporter gene activation. HEK293T cells were transfected with a reporter construct containing the IFN-α4 promoter. Cells were co-transfected with 50 ng IRF7 or empty vector (EV) control and increasing amounts of (a) Ro52-expressing construct or (b) a plasmid expressing a Ro52 mutant lacking the RING-finger domain. Cells were assayed for reporter gene activity 18 hrs post-transfection. *, *p*<0.01 as determined by Student's *t* test. NS, not significant.

Previous work by Espinosa *et al* has clearly shown that TLR7- and TLR9-mediated gene induction is significantly altered in Ro52-deficient mice. Specifically both IFN-α and IFN-β were enhanced in splenocytes derived from Ro52-deficient mice compared with wild-type controls following infection with herpes simplex virus (HSV)-2, strongly suggesting that Ro52 is a negative regulator of IRF7 activity [26]. To test this hypothesis and to determine the effect of Ro52 on IRF-mediated IFN production, HEK293T cells were transfected with plasmids expressing IRF7, the IFN-α promoter and increasing amounts of Ro52. Whilst transfection of cells with an empty control vector did not drive IFN-α promoter activity, transfection with a plasmid expressing IRF7 resulted in efficient stimulation of the IFN-α promoter (Fig. 3A). The addition of increasing concentrations of a plasmid expressing Ro52 significantly inhibited IRF7-driven IFN-α promoter activity in a dose-dependent manner. In contrast, increasing amounts of a plasmid expressing a mutant Ro52 lacking the really interesting new gene (RING)-finger domain, required for the E3 ligase activity of Ro52, failed to significantly inhibit IRF7-driven IFN-α promoter activity (Fig. 3B). Taken together, these results confirm that the observed inhibition of the IFN-α4 promoter is mediated by full-length active Ro52, indicative of a role for Ro52 as a negative regulator of IRF7-driven type I IFN production.

### Ro52 negatively regulates IRF7 expression by initiating IRF7 degradation

**Figure 4:**
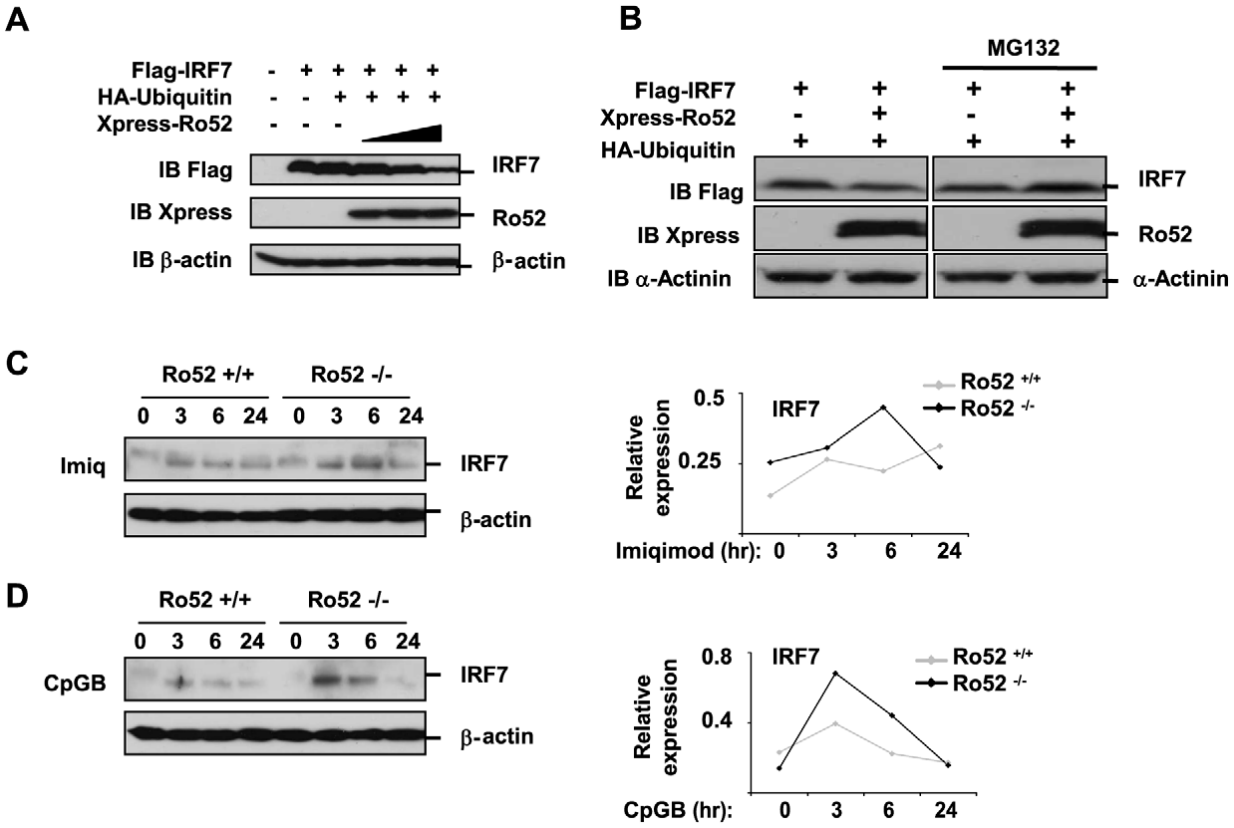
Ro52 mediates its effects by destabilising IRF7. (A) HEK293T cells were transfected with 500 ng of Flag-IRF7, 1 µg of HA-ubiquitin and increasing amounts of Xpress-Ro52 (500 ng, 1 µg and 2 µg) for 18 hrs. Presence of Flag-IRF7, HA-ubiquitin, Xpress-Ro52 and β-actin was detected by immunoblotting. (B) HEK293T cells were transfected with 500 ng of Flag-IRF7, 1 µg of HA-ubiquitin and 500 ng of Xpress-Ro52 for 18 hrs. Transfected cells were treated with either MG132 or DMSO for 2 hours prior to cell lysis. Presence of Flag-IRF7, Xpress-Ro52 and α-actinin was detected by immunoblotting. (C and D) Bone marrow-derived macrophages from wildtype and Ro52-deficient mice were stimulated with either (C) 10 µg/ml imiquimod or (D) 3 µM CpG-B at the indicated timepoints. Expression of IRF7 and β-actin were detected by immunoblotting. IRF7 expression was quantitated by densitometry following normalisation to β-actin expression as indicated in accompanying graphs.

To determine the mechanism by which Ro52 negatively regulates the IFN-α4 promoter we examined the fate of IRF7 following association with Ro52. HEK293T cells were transfected with plasmids expressing Flag-IRF7, HA-ubiquitin and increasing Xpress-Ro52. In this experiment, in contrast to that shown in Figure 2, the amount of Flag-IRF7 expressing plasmid transfected into the cells was significantly reduced in order that the effect of Ro52 on IRF7 fate might be observed. Cell lysates were immunoblotted for Flag-IRF7 and, following a short exposure time, Ro52-induced IRF7 degradation was observed, an effect which was substantially increased at the highest concentration of Ro52 (Figure 4A). This result indicates that Ro52 targets IRF7 for polyubiquitination in order to induce degradation of the transcription factor post-TLR stimulation. Furthermore, this Ro52-mediated degradation of IRF7 was inhibited in the presence of MG132, a proteasome inhibitor, indicating that IRF7 is targeted to the proteasome for degradation (Figure 4B).

In order to investigate the effects of Ro52 deficiency on IRF7 levels in primary immune cells, we used bone-marrow derived macrophages (BMDMs) from Ro52-deficient mice, which display enhanced type I IFN production following TLR stimulation [26]. As IRF7 is a key transcription factor which regulates the expression of type I IFNs, we analyzed IRF7 expression following TLR7 and TLR9 stimulation in BMDMs from wildtype and Ro52-deficient mice. As shown in Figure 4C and 4D, in wild type mice, IRF7 was induced following 3 hours stimulation with either imiquimod (Figure 4C) or CpG-B (Figure 4D). However, in Ro52-deficient mice a substantial increase in IRF7 induction was observed, which peaked at 6 hrs following imiquimod stimulation and at 3 hrs following CpG-B stimulation. This result is consistent with the Ro52-mediated ubiquitination and degradation observed in the HEK293T cells, and suggests that in the absence of Ro52, IRF7 levels accumulate following TLR-7 and TLR-9 stimulation, thus contributing to the enhanced IFN responses observed in the knockout mice [26]. Taken together, these findings indicate that Ro52 functions to limit IRF7 induction following TLR7 and TLR9 activation, similar to the previously described role for Ro52 in the negative regulation of IRF3 following TLR3 and TLR4 activation and consistent with the reported effects of Ro52 deficiency in mice.

## Discussion

Recent work has demonstrated that loss of Ro52 can lead to tissue inflammation and systemic autoimmunity through activation of the IL-23/TH17 axis and has clearly established Ro52 as an important negative regulator of proinflammatory cytokine and type I IFN production (26). Work prior to this had demonstrated that Ro52 could interact with IRF3 and IRF8 and regulate the stability levels of these transcription factors through the ubiquitin-proteasome system [13], [27]. We observed in an earlier study that Ro52 associates with overexpressed IRF7 and speculated that Ro52 might regulate this transcription factor and other IRF family members [13]. We now present evidence identifying Ro52 as an endogenous E3 ligase that negatively regulates IRF7 expression. Endogenous immunoprecipitation of Ro52-bound proteins revealed that IRF7 associates with Ro52, an effect which increases following TLR7 and TLR9 stimulation. This suggests that Ro52 interacts with IRF7 post-pathogen recognition and may function as a regulator of IRF7 stability, similarly to its role in regulating IRF3 [13].

Regulation of IRF7 stability is a potent mechanism of controlling type I IFN production in response to viral infection and has previously been shown to be highly cell specific [19]. Several viral proteins have been identified which encode E3 ligases and promote IRF7 degradation via the ubiquitin-proteasome system. The Kaposi's sarcoma-associated herpesvirus (KSHV)-encoded immediate-early replication and transcription activator (RTA) protein targets IRF7 for proteasomal degradation [12], as does the rotaviral non-structural protein 1 [9]. The function of these viral proteins is presumably to inhibit the anti-viral response by degrading IRF7; however the endogenous E3 ligase responsible for degrading IRF7 as a protective measure against the harmful effects of type I IFN overproduction has remained elusive to date. Our data indicates that Ro52 ubiquitinates IRF7, adding both monoubiquitin and polyubiquitin chains to the transcription factor. A previous study identified that degradation of IRF7 was ubiquitin-dependent, through use of a dominant-negative form of the Skp, Cullin, F-box (SCF) ubiquitin ligase complex protein Cul1 [19]. Accordingly, we observed a dose-dependent decrease in IRF7 levels when expressed with constant ubiquitin and increasing amounts of Ro52 indicating that, like Cul1, Ro52 promotes degradation of IRF7.

Ro52-deficient mice, following tissue injury, develop symptoms including severe dermatitis, systemic lupus with hypergammaglobulinemia and autoantibodies to DNA, indicating that a loss of Ro52 can cause a lupus-like disease [26]. We observed a substantial increase in IRF7 induction in bone marrow-derived macrophages from Ro52-deficient mice stimulated with both imiquimod and CpG-B, when compared to wildtype mice. This result is consistent with a role for Ro52 in the negative regulation of IRF7 signalling. Thus, it seems likely that whilst TRAF6-mediated monoubiquitination activates IRF7, Ro52-mediated polyubiquitination promotes the degradation of IRF7 following TLR7 and TLR9 stimulation.

This novel role for Ro52, in negatively regulating IRF7 stability, may provide a mechanism to protect the host from the overproduction of type I IFNs, a contributing factor to the pathogenesis of systemic lupus erythematosus [28]. This hypothesis is supported by the ability of Ro52 to dose-dependently inhibit luciferase activation by the IFN-α4 promoter when stimulated with IRF7. In addition, bone marrow-derived macrophages from Ro52-deficient mice have previously been shown to produce increased levels of type I IFNs and proinflammatory cytokines following stimulation with TLR9 ligands [26], an effect which can now be explained by the negative regulation of IRF7 signalling by Ro52. Several studies have shown that Ro52 plays a dual role in regulating IRF family member stability [13], [26], [27], [29], [30]. These observations, along with the results presented here, suggest that Ro52 has a complex role in regulating innate immune responses, and may be involved in both the activation and degradation of IRF family members. In addition, regulation of IRF7 is not solely controlled by the classical ubiquitin-proteasome system, as recent studies have shown a role for SUMOylation [31] and the translational repressors 4E-BP1 and 4E-BP2 [32] in inhibiting IRF7 signalling. It is likely that Ro52 lies downstream of both TLRs and the RLRs such as RIG-I, similar to its role in regulating IRF3 stability [13]. The precise mechanism by which the activity of Ro52 is regulated downstream of both TLRs and RLRs is currently being investigated in our lab.

In summary, we provide evidence that Ro52 functions to degrade IRF7 following TLR7 and TLR9 stimulation. These receptors are nucleic acid-sensing TLRs known to be involved in the pathogenesis of autoimmune diseases such as systemic lupus erythematosus. In addition, it has been postulated by us and others that Ro52, an IFN-inducible ubiquitin ligase, is central to a negative feedback loop which protects the host from prolonged exposure to type I IFNs [33]. This is consistent with both the established role of Ro52 in negatively regulating IRF3 expression post TLR3 and TLR4 stimulation and the novel role of Ro52, reported here, in ubiquitinating and degrading IRF7. Taken together with reports of Ro52 regulating other IRF family members, both positively and negatively, Ro52 is emerging as a multifunctional protein with a critical role in regulating innate immune responses. Further studies may identify approaches to manipulate Ro52 activity, leading to potential therapeutic strategies to help prevent autoimmune disease.

## Materials and Methods

### Cell culture

HEK293T cells and HeLa cells were cultured in Dulbecco's modified Eagle's medium (DMEM) supplemented with 10% foetal calf serum (FCS) and 10 µg/ml gentamicin. The generation of Ro52 knockout mice has been described previously [26]. Bone marrow-derived macrophages were harvested from femurs obtained from wildtype and Ro52-deficient mice and cultured in DMEM supplemented with 10% FCS, 10 µg/ml gentamicin and 10–15% L929 supernatant for 6–8 days prior to being stimulated with imiquimod or CpG-B/IFN-γ.

### Plasmids and reagents

Flag-tagged IRF7, pEF-Bos-TRIF-Flag and the IFNα4 promoter-luciferase constructs were from Dr. Kate Fitzgerald (University of Massachusetts Medical School, Worcester, MA). Wildtype and ΔExon1 Xpress-tagged Ro52 constructs were provided by Dr. David Rhodes (Cambridge Institute for Medical Research, Cambridge, UK) and HA-ubiquitin, HA-ubiquitin R48K, and HA-ubiquitin R63K plasmids were from Dr. Andrew Bowie (School of Biochemistry with Immunology, Trinity College Dublin, Ireland). Primary antibodies used were anti-HA, anti-IRF7 (both Santa Cruz Biotechnologies), anti-Flag (Sigma), anti-Xpress™ (Invitrogen), α-actinin (Santa Cruz Biotechnologies) and anti-β-actin (Abcam). Polyclonal antibodies against Ro52 that were used in immunoprecipitation experiments were made by Sigma-Genosys and anti-Ro52 antibodies used in Western blots were purchased from BioReagents, Cambridge, UK.

### Luciferase reporter gene assays

HEK293T cells were transfected with an IFNα4 promoter-luciferase construct, an IRF7 construct (50 ng) and increasing amounts of a wildtype or ΔExon1 Ro52 construct (10 ng, 50 ng and 100 ng). All transfections were performed using Metafectene™ (Biontex) according to the manufacturer's recommendations. Following transfection, luciferase activity was standardized to *Renilla* luciferase plasmid activity to normalize for transfection efficiency. Each luciferase is representative of three independent experiments.

### Immunoprecipitation

Cells were lysed on ice in 1× radioimmune precipitation lysis buffer (1× PBS, 1% Nonidet P-40, 0.5% Na-deoxycholate, 0.1% SDS, 1 mM KF, 1 mM Na_3_VO_4_, 10 µg/ml leupeptin and 1 mM phenylmethylsulfonyl fluoride) followed by immunoprecipitation with either anti-HA agarose, or anti-Flag or anti-Ro52 bound to protein-G sepharose beads, as indicated in the figure legends. Immunoprecipitates were analysed by Western blot. Each blot is representative of up to three independent experiments.
